# Analysis of sweating efficiency and its effects on the heat strain alleviation of clothed subjects

**DOI:** 10.14814/phy2.14694

**Published:** 2021-01-13

**Authors:** Kouhei Kuwabara, Yasuhiro Hamada, Hideki Kubota

**Affiliations:** ^1^ Department of Creative Engineering National Institute of Technology Kushiro College Kushiro Japan; ^2^ Graduate School of Engineering Hokkaido University Sapporo Japan; ^3^ Graduate School of Engineering Hokkaido University, Retired Muroran Japan

**Keywords:** heat strain, regional evaporative capacity, regional sweat rate, skin temperature, skin wetness, sweating efficiency

## Abstract

Sweating efficiency (SE) is essential for evaluating heat strain. The dripping of sweat off the skin surface of a nude subject occurs locally at an area where the secreted sweat exceeds the local evaporative capacity. However, in clothed subjects, “dripping” sweat is absorbed by clothing. In the present paper, the cooling efficiency of the sweating of a clothed subject is analyzed in relation to SE. First, typical patterns for the regional distribution of the sweat rate (SR) and the capacity of evaporation (CE) of a nude subject were introduced, and the dripping sweat rate was derived as a surplus of the SR over the CE; an equation of SE was derived from combinations of the two typical SR patterns and the uniform CE pattern. Then, the values of SE were calculated numerically, and the results were found to be approximately equal to those obtained experimentally by Alber–Wallerström & Holmér and theoretically from the equation of 1 − 0.5*w_sw_* ^2^ used in ISO7933. Based on these results, the SE was improved by arranging the distribution of the CE by controlling air velocities over the body surface. Further, the improved SE was found to contribute to the heat strain alleviation of clothed subjects.

## Graphical abstract

We analytically estimated the sweating efficiency of humans based on the surplus of the regional sweat rate over the regional capacity of evaporation. We improved the sweating efficiency by exposing the higher air velocity to the trunk area and proved its effect on heat strain alleviation of a clothed subject by predicting the mean skin temperature.

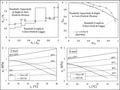

## 1 INTRODUCTION

The sweating efficiency (SE), or the evaporative efficiency of sweating, is of prime importance for estimating human response to a hot environment. It is essential for assessing heat strain to estimate the amount of heat loss accurately through the evaporation of sweat (Kubota et al., 2014a, 2014b); however, it requires time‐consuming work to elucidate the SE by experiments. Until now, a limited number of experiments with human subjects have been conducted to identify the SE. Candas et al. (1979a, 1979b) measured the amount of dripping sweat of male subjects in a prone position, and it showed the relationship between skin wettedness and SE. The effects of heat acclimation and wind speeds on SE were examined. In addition, the values of SE were derived quantitatively by dividing the human body into three parts with relevant local evaporative heat transfer coefficients, where local sweat rates were assumed to be uniform over the entire body; it was discussed that the discrepancies of the SE values between those by experiments and those by calculations were attributable to the local sweating rate differences (Candas et al., 1979a). Alber & Holmér (1994); Alber‐Wallerström & Holmér (1985) measured the amount of dripping sweat of male and female subjects during exercise with a bicycle ergometer and showed the relationship between skin wettedness and SE. The SE values obtained by Candas et al. and Alber and Holmér differed greatly, which may have been caused by variations in the regional distribution of the sweat rate (SR), capacity of evaporation (CE), metabolic rate, and postures. However, no quantitative considerations were presented. Meanwhile, in clothed subjects, “dripping” sweat is absorbed by clothing, and it evaporates from the surface of the wetted clothing; thus, the SE described above is only related to nude subjects. Havenith et al. (2008) performed a thermal sweating mannequin study on the evaporative cooling efficiency (not evaporative efficiency) of clothed subjects using clothing with different levels of vapor permeability. No relation to the SE was given. Though such an experiment has the potential for investigating the SE, there would be a limitation on the mannequin's movement such as high‐speed walking.

In the present study, we first analyze the SE based on the relationship between the regional SR and regional CE focusing on exercising subjects. Many studies were performed on SR (Kuno, 1934; Smith & Havenith, 2011; Weiner, 1945) and CE (Nishi & Gagge, 1970; Oliveira et al., 2014), where the CE is evaluated based on the convective heat transfer coefficients. However, to our knowledge, no attempt has been made to consider the SE quantitatively in relation to the regional SR and CE. The ineffective sweat rates of the dripping off the skin surface are derived as the surplus of SR over the CE. Based on these rates, the SE is analytically obtained for a nude subject and compared with that obtained by experimental data and equation 1 − 0.5*w_s_*_w_^2^ (*w_sw_*: wetness) applied in ISO7933 (ISO^7933^, (1989)). For the analysis, typical experimental data on the SR and CE available in the literature are used (Appendix A).

Based on these results, we improve the SE by arranging the distribution of the CE by controlling air velocities over the body surface. Then, we predict the mean skin temperature (MST) as a typical heat strain indicator of a clothed subject using a previously developed human model (Kubota et al., 2014b) and applying the improved SE. The results are compared with those obtained from 1 − 0.5*w_s_*_w_^2^, which can provide insight into ways of reducing heat‐associated risks (Kubota et al., 2014b) and designing and operating a sweating mannequin. A list of abbreviations and symbols is given below.

## 2 METHODS

### 2.1 Assumption on the dripping of sweat

The dripping of sweat of a nude subject occurs locally at an area where the secreted sweat exceeds the local evaporative capacity (Kerslake, 1972). The secreted sweat from the sweat glands flows through the skin surface and spreads over the wet area until the rate of evaporation from the wet surface becomes equal to that of sweat. The dripping of sweat begins when the wet area reaches the maximum area of the gland (i.e., the boundary between adjacent glands); the surplus of the SR over the CE accumulates on the skin surface and drips off when gravity overcomes the surface tension. In practice, the dripping sweat—ineffective sweat—can flow down along the skin surface and shift to the adjacent area of the body surface where some portion could evaporate and transform the effective sweat. In the present paper, we neglected this effect, as it could lead to overestimating the dripping SR, that is, underestimation of the SE. Hence, the authors incline toward a slight underestimation of the SE in our models.

### 2.2 Regional sweat rate

**FIGURE 1:**
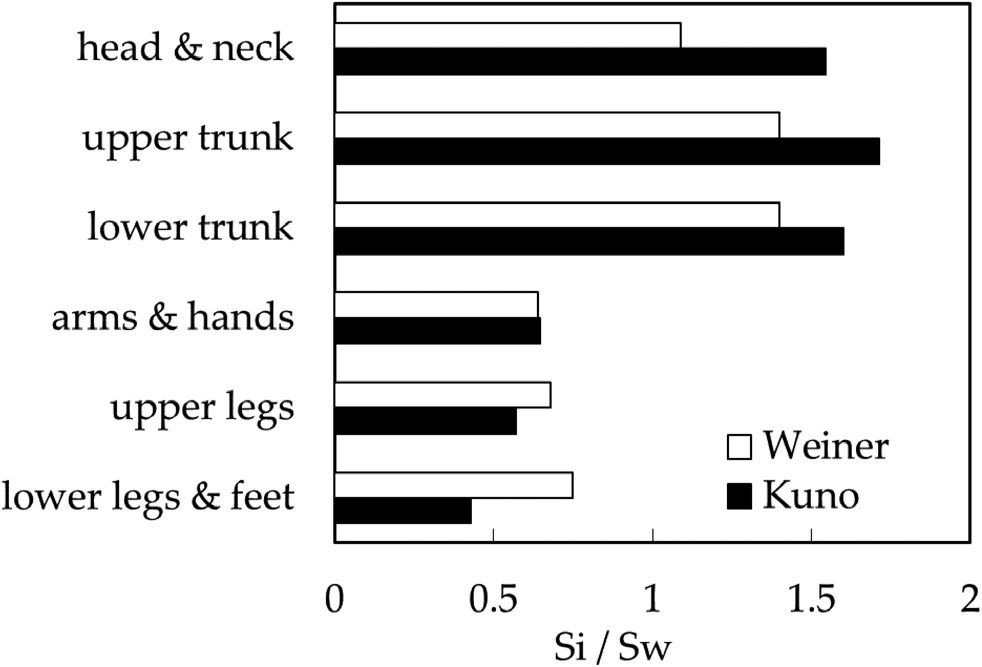
Normalized regional sweat rate *S_i_*/*S_w_* (SR) at typical sections of the body given by Kuno (1934) and Weiner (1945) (Data modified by the authors).

**FIGURE 2:**
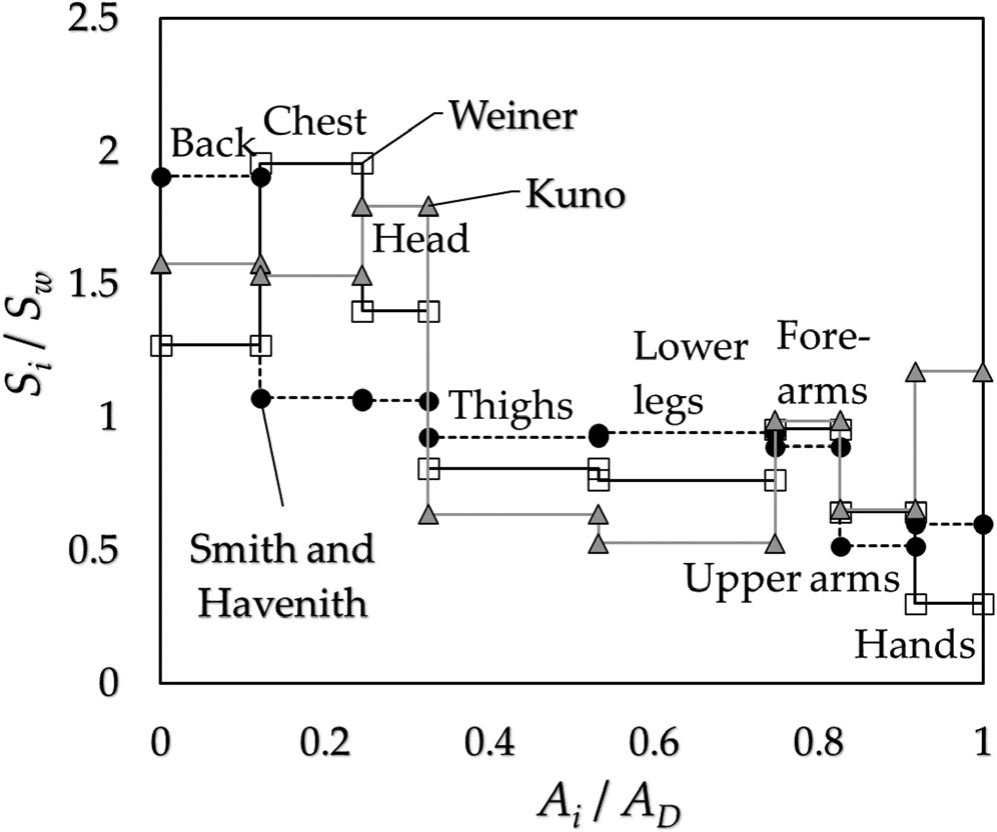
The SR (*S_i_*/*S_w_*) at 8 regions of the body surface are presented against the body surface area (*A_i_*/*A_D_*). The original data were given by Kuno (1934) (gray square), Weiner (1945) (□), and Smith & Havenith (2011) (●). (Data modified by the authors)

During the classification of the distribution patterns of the regional SR over the body surface, two categories arise:

1. Two‐part‐pattern: The SR arranged for the typical regions of the body is shown in Figure 1 (Kuno, 1934; Weiner, 1945), where the data (= *S_i_* in W∙m^−2^) are shown in the ratio of *S_i_* over that of the mean value *S_w_*, *S_i_*/*S_w_*. As it is apparent at first glance, the body surface appears to be divided into two parts as a stepwise: the high (head and trunk) and low (limbs) SR parts.
2. Eight‐step‐pattern: The values of CE are measured at the typical sections of the body surface as follows: 1. back, 2. chest, 3. head, 4. thighs, 5. lower legs, 6. upper arms, 7. forearms, 8. hands. These eight sections are used to calculate the surplus of the ineffective dripping sweat rate and organize the data of SR in corresponding sections. The typical 8‐step‐pattern of SR for exercising subjects is shown in Figure 2 based on the existing data (Kuno, 1934; Smith & Havenith, 2011; Weiner, 1945), which were used in the present work for the analysis.

### 2.3 Regional capacity of evaporation

**FIGURE 3:**
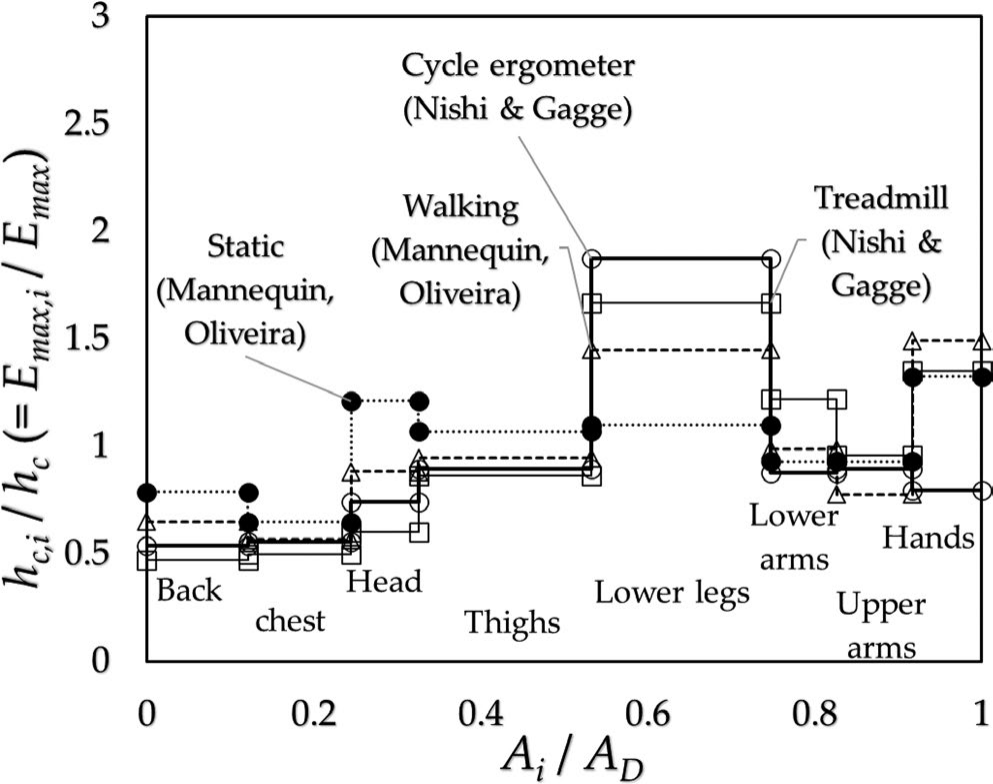
Regional convective heat transfer coefficients in units of *h_c_*_,*i*_/*h_c_* ≈ *E_max_*_,*i*_/*E_max_* (CE); original data were given by Nishi & Gagge (1970) (○: Cycle‐ergometer 60 rpm, □: Treadmill 3 mph), Oliveira et al. (2014) (△:Walking 45 steps/min, ●: Static (Standing still))

The value of CE (*E_max_*_,_ *_i_*) is evaluated based on the convective heat transfer coefficient (*h_c_*_,_ *_i_*) for a nude subject and is given as *E_max_*_,_ *_i_*/*E_max_* = *h_c_*_,_ *_i_*/*h_c_* (*E_max_* and *h_c_* are the mean values) (Appendix B). Nishi & Gagge (1970) measured the values of *h_c_*_,_ *_i_* at eight regions (10 points) of the body surface of a subject: 1. back, 2. chest, 3. head, 4. upper legs, 5. lower legs and feet, 6. lower arms, 7. upper arms, and 8. hands in the descending order for the amount of SR (Smith & Havenith, 2011). The typical regional distributions of *h_c_*_,_ *_i_*/*h_c_* are shown in Figure 3; the data were given by Nishi & Gagge (1970) (cycle ergometer at 60 rpm, treadmill exercises at 3 mph) and Oliveira et al. (2014) (mannequin with walking movement at 45 steps/min and standstill).

For the analysis of SE, it is appropriate to use the data of SR and CE of the subject performing the same kind of exercise. In the case of the SR data given by Smith & Havenith, the SRs were obtained on a treadmill exercise under 2 m/s frontal air velocity, and those by Weiner, which were acquired for subjects stepping on and off a stool, the corresponding CEs were not available. The present authors estimated the values of CE from the data given by Oliveira et al. and Nishi & Gagge (Appendix C).

To determine the characteristics of SE, we introduced an imaginary uniform pattern for the regional distribution of CE in addition to the 8‐step‐pattern of the measured region. The uniform‐pattern provides the SE for an extreme condition of the unevenness of the CE distribution.

### 2.4 Sweating efficiency and skin wetness

The dripping of sweat occurs locally; in the analysis performed for the two‐part and eight‐step patterns, we considered that “the locally secreted sweat” is identical to the average SR for each region.

The SE, *η_sw_*, and skin wetness, *w_sw_*, are defined as

(1) $$\eta_{\mathrm{sw}}=\frac{\left(S_{w}‐S_{\mathrm{dr}}\right)}{S_{w}}$$

(2) $$w_{\mathrm{sw}}=\frac{S_{w}‐S_{\mathrm{dr}}}{E_{\max}}$$

where *S_dr_* denotes the dripping sweat rate of the whole body in W∙m^−2^, and *S_w_* and *E_max_* represent the mean values of SR and CE, respectively, in W∙m^−2^; *η_sw_* and *w_sw_* are non‐dimensional.

Then, *w_sw_* is rewritten as

(3) $$w_{\mathrm{sw}}=\eta_{\mathrm{sw}}w_{v}$$

where *w_v_* denotes the virtual skin wetness

(4) $$w_{v}=\frac{S_{w}}{E_{\max}}$$

**FIGURE 4:**
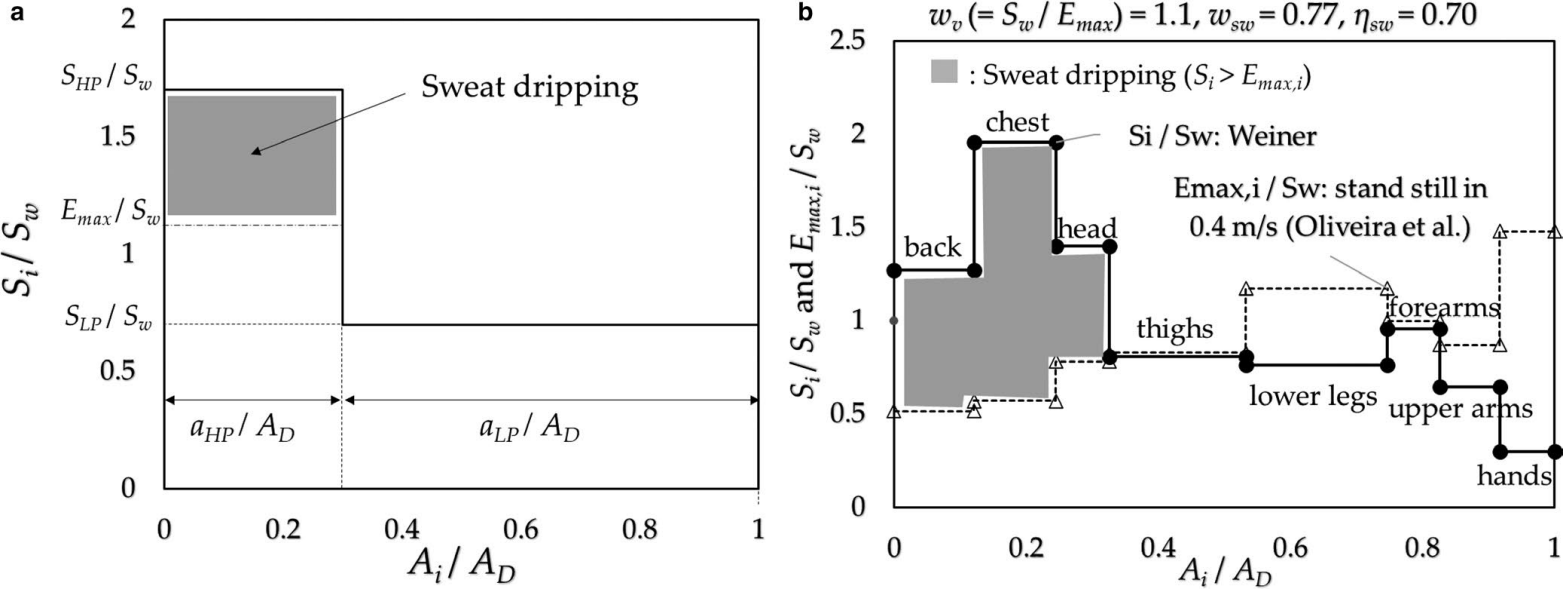
Schematic view of SR patterns of the: (a) two‐part‐pattern; (b) the relationship between SR (*S_i_*/*S_w_*) by Weiner and CE (*E_max_*_,*i*_/*S_w_*) (for standing still in a 0.4 m/s air flow using data by Oliveira) at *w_v_* = 1.1. The dripping of sweat occurs at the region of *S_i_*/*S_w_* > E_max,*i*_/*S_w_*, *A_i_*/*A_D_* ≤0.3; i.e., the back, chest and head, and forearms are wet. The SE was 0.70 (*S_dr_*/*S_w_* = 0.30) and *w_sw_* = 0.77

The dripping sweat rate *S_dr_* is obtained by summing up the regional dripping SR *S_dr_*_,_ *_i_*, which is given at the point where *S_i_* > *E_max_*_,_ *_i_*. As shown schematically in Figure 4a,b for the simplified pattern and 8‐step‐pattern models,

(5) $$S_{dr,i}=\frac{\left(S_{i}‐E_{\max,i}\right)a_{i}}{A_{D}}$$

where *S_i_* and *E_max_*_,_ *_i_* are SR and CE at a region *i*, respectively, in W∙m^−2^, and *a_i_* and *A_D_* denote the body surface area of region *i* and DuBois body surface area, respectively, in m^2^.

This is rewritten as

(6) $$S_{dr,i}=S_{w}\left(\frac{S_{i}}{S_{w}}‐\frac{E_{\max,i}}{E_{\max}}\frac{E_{\max}}{S_{w}}\right)\frac{a_{i}}{A_{D}}=S_{w}\left(\frac{S_{i}}{S_{w}}‐\frac{E_{\max,i}}{E_{\max}}\cdot \frac{1}{w_{v}}\right)\frac{a_{i}}{A_{D}}$$

The values of *a_i_*/*A_D_*, *S_i_*/*S_w_*_,_ and *E_max_*_,_ *_i_*/*E_max_* are shown in Figures 2 and 3.

When the value of *w_v_* (= *S_w_*/*E_max_*) is given, *S_dr_*_,_ *_i_* is obtained as

(7) $$\frac{S_{\mathrm{dr}}}{S_{w}}=\frac{\sum S_{dr,i}}{S_{w}}=\sum \left(\frac{S_{i}}{S_{w}}‐\frac{E_{\max,i}}{E_{\max}}\cdot \frac{1}{w_{v}}\right)\frac{a_{i}}{A_{D}}\;\text{for}\;\frac{S_{i}}{S_{w}}>\frac{E_{\max,i}}{E_{\max}}\cdot \frac{1}{w_{v}}$$

The sweating efficiency (SE, *η_sw_*) is given as

(8) $$\eta_{\mathrm{sw}}=1‐\sum \left(\frac{S_{i}}{S_{w}}‐\frac{E_{\max,i}}{E_{\max}}\cdot \frac{1}{w_{v}}\right)\frac{a_{i}}{A_{D}}\;\mathrm{for}\;\frac{S_{i}}{S_{w}}>\frac{E_{\max,i}}{E_{\max}}\cdot \frac{1}{w_{v}}$$

Note that the skin wetness *w_sw_* differs from the skin wettedness *w* introduced by Gagge (Gagge, 1937) and defined as

(9) $$w=w_{\mathrm{sw}}+\frac{\left(1‐w_{\mathrm{sw}}\right)E_{\mathrm{dif}}}{E_{\max}}$$

*w* ≈ 0.06 + 0.94*w_sw_* (ASHRAE 2009 (ASHRAE, 2009)). We assumed *w* ≈ *w_sw_*.

## 3 RESULTS

### 3.1 Imaginary SE for uniformly distributed CE and two‐part‐pattern SR

The equation for SE is derived as

The mean sweat rate *S_w_* in W∙m^−2^ is

(10) $$S_{w}=\frac{\left(S_{\mathrm{HP}}a_{\mathrm{HP}}+S_{\mathrm{LP}}a_{\mathrm{LP}}\right)}{A_{D}}$$

where *a_HP_* and *a_LP_* are the skin surface areas of the high and low SR parts, respectively, in m^2^; *S_HP_* and *S_LP_* denote the sweat rates at the high and low SR parts, respectively, in Wm^−2^.

The value of *S_dr_* under the conditions between *S_HP_* or *S_LP_* and *E_max_* is given as

A: *S_HP_* ≤ *E_max_* (No sweat drips.)

(11) $$S_{\mathrm{dr}}=0,$$

B: *S_HP_* > *E_max_* ≥ *S_LP_* (Sweat drips at trunk/head.) (Figure 4a)

(12) $$S_{\mathrm{dr}}=\frac{\left(S_{\mathrm{HP}}‐E_{\max}\right)a_{\mathrm{HP}}}{A_{D}},$$

C: *S_LP_* > *E_max_* (Sweat drips and skin is fully wet.)

(13) $$S_{\mathrm{dr}}=\frac{\left(S_{\mathrm{HP}}‐E_{\max}\right)a_{\mathrm{HP}}+\left(S_{\mathrm{LP}}‐E_{\max}\right)a_{\mathrm{LP}}}{A_{D}},$$

For the area *S_HP_* > *E_max_* ≥ *S_LP_*, by substituting Eq. (12) into Eq. (1), we get

(14) $$\eta_{\mathrm{sw}}=\frac{\left(S_{w}‐S_{\mathrm{dr}}\right)}{S_{w}}=1‐\left(\frac{a_{\mathrm{HP}}}{A_{D}}\right)\frac{\left(S_{\mathrm{HP}}‐E_{\max}\right)}{S_{w}}$$

As shown in Eq. (14), the equation for *η_sw_* is expressed in the form (Kubota et al., 2014a)

(15) $$\eta_{\mathrm{sw}}=a_{\mathrm{dr}}+\frac{b_{\mathrm{dr}}}{w_{v}}$$

where $a_{\mathrm{dr}}=1‐\frac{a_{\mathrm{HP}}}{A_{D}}\frac{S_{\mathrm{HP}}}{S_{w}}=\frac{a_{\mathrm{LP}}}{A_{D}}\frac{S_{\mathrm{LP}}}{S_{w}},\;b_{\mathrm{dr}}=\frac{a_{\mathrm{HP}}}{A_{D}}$.

By applying Eq. (3), this is rewritten as

(16) $$\begin{array}{c} w_{\mathrm{sw}}=\eta_{\mathrm{sw}}w_{v}=\left(a_{\mathrm{dr}}+\frac{b_{\mathrm{dr}}}{w_{v}}\right)w_{v}=a_{\mathrm{dr}}w_{v}+b_{\mathrm{dr}}\\ w_{v}=\frac{w_{\mathrm{sw}}‐b_{\mathrm{dr}}}{a_{\mathrm{dr}}}\\ \eta_{\mathrm{sw}}=a_{\mathrm{dr}}\left(1+\frac{b_{\mathrm{dr}}}{w_{\mathrm{sw}}‐b_{\mathrm{dr}}}\right) \end{array}$$

The following relations are given.

(17) $$\frac{S_{\mathrm{HP}}}{S_{w}}=\frac{\left(1‐a_{\mathrm{dr}}\right)}{b_{\mathrm{dr}}},\;\frac{S_{\mathrm{LP}}}{S_{w}}=\frac{a_{\mathrm{dr}}}{\left(1‐b_{\mathrm{dr}}\right)},$$

The parameters were determined from experimental results presented by Alber‐Wallerström & Holmér (1985), (cycle ergometer) and by Candas et al. (1979a), (prone), as

[Cycle ergometer]: *a_dr_* = 0.37, *b_dr_* = 0.31 = *a_HP_*/*A_D_*, *S_HP_*/*S_w_* = 2.0, *S_LP_*/*S_w_* = 0.54, *S_LP_*/*S_HP_* = 3.7

[Prone]: *a_dr_* = 0.36, *b_dr_* = 0.48 = *a_HP_*/*A_D_*, *S_HP_*/*S_w_* = 1.3, *S_LP_*/*S_w_* = 0.69, *S_LP_*/*S_HP_* = 1.9

**FIGURE 5:**
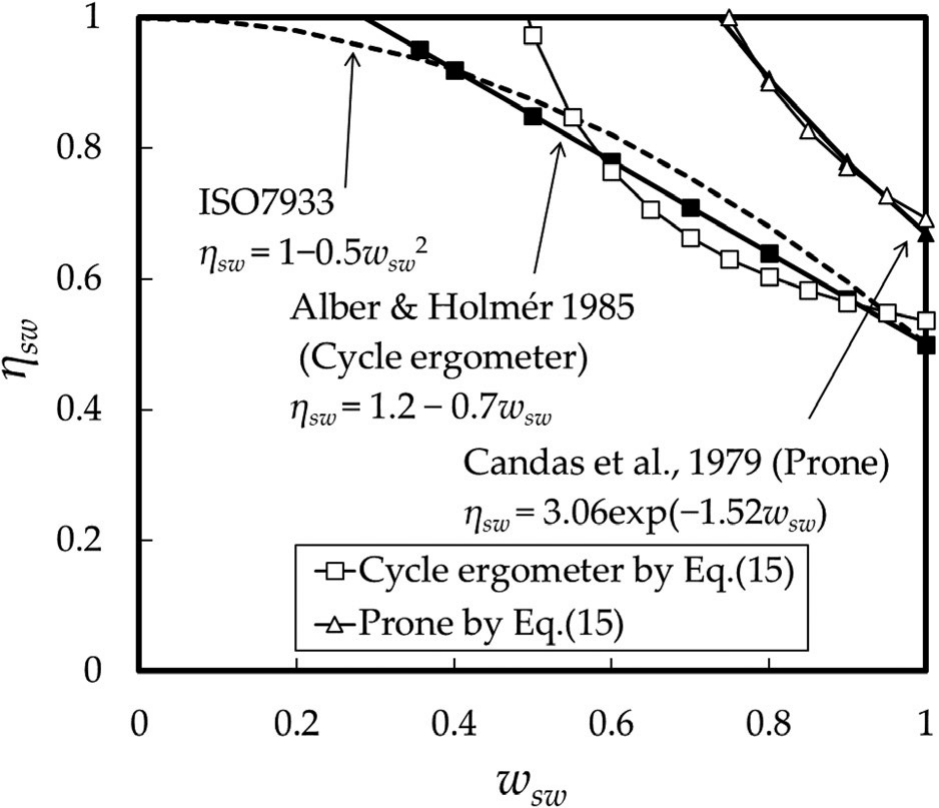
Relationship between the SE (*η_sw_*) and skin wetness, *w_sw_*. Results obtained from Eq.(15) for the 2‐part‐pattern for subjects doing cycle‐ergometer exercise (□) and in the prone posture (△); for *η_sw_* = 1.2 − 0.7*w_sw_* by Alber & Holmér (■) (Alber‐Wallerström & Holmér, 1985), *η_sw_* =*3*.06exp(−1.52*w_sw_*) by Candas et al. (filled triangle) (Candas et al., 1979a), and *η_sw_* = 1 ‒ 0.5*w_sw_* ^2^ as in ISO7933 (dashed curved).

The results obtained from Eq. (15) are shown in Figure 5 (Kubota et al., 2014a) and compared with the results by Alber & Holmér, Candas, and by *η_sw_* *=_1_* – 0.5*w_sw_^2^* in ISO7933 (ISO^7933^, ^1989^).

### 3.2 Computing SE based on SR(S_i_) and CE (E_max,i_) data at eight regions—eight‐step‐pattern

The values of SE were calculated for a subject participating in the following activities: (a) exercising on a cycle ergometer; (b) standing still (Kuno, 1934); (c) stepping on and off a stool (Weiner, 1945); and (d) running on a treadmill (Smith & Havenith, 2011). The procedure was applied as described in Section 2.4 to the SR and CE data arranged into eight regions of the body surface‐eight‐step‐pattern.

(a) The SE of a subject exercising on a cycle ergometer.

**FIGURE 6:**
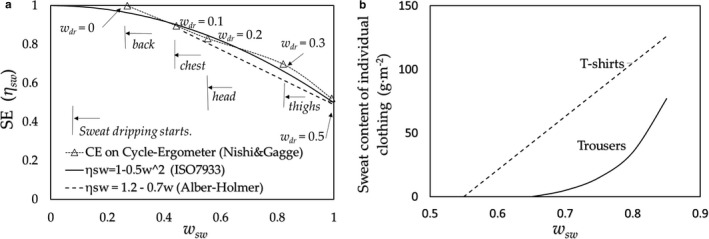
(a) SE calculated for the cycle‐ergometer exercise, the CE by Nishi & Gagge with the SR by Smith & Havenith (△); (b) Sweat content of individual clothing after 2 hours of cycle‐ergometer exercise in a hot environment with subjects wearing T‐shirts and trousers, which demonstrates the relationship between wetness and *wdr* shown in (a), where *w_dr_* ≈ 0.1 corresponds to the back area, *w_dr_* ≈ 0.3

We deployed the distribution of the CE of a subject exercising on a cycle ergometer given by Nishi & Gagge (1970) (Figure 3) combining with the data of SR on a subject running on a treadmill given by Smith & Havenith (2011) (Figure 2), where we assumed that the distribution of SR on a subject exercising on a cycle ergometer is almost the same with that running on a treadmill. The results are shown in Figure 6a with the experimental results obtained by Alber‐Wallerström & Holmér (1985) and equation 1 – 0.5*w_sw_* ^2^ **;** the results of the SE provide the following quantitative information:

1. At *w_sw_*≈ 0.25, the dripping of sweat begins;
2. At *w_sw_* ≈ 0.25–0.45, *w_dr_* (= *A_dr_*/*A_D_*) reaches approximately 0.1, which corresponds to the area of the “*back*,” that is, only the skin surface of the *back* is wet. (As an exception, the part of the forehead will get wet as it is one of the highest SR spots.)
3. At *w_sw_* ≈ 0.55, *w_dr_* becomes approximately 0.2, which means that the *chest* and *head* also get wet. The condition holds until *w_sw_* ≈ 0.8, that is, the dripping of sweat occurs at the low CE part of the trunk;
4. At *w_sw_* ≿ 0.8 (*w_dr_* ≈ 0.5), the sweat dripping occurs at the region of the *thighs*/*pelvic* parts, that is, when *w_sw_* exceeds approximately 0.8, the dripping of the sweat at the lower half of the body starts.

These conclusions are confirmed by the experiments of the 2‐hour‐cycle ergometer exercises conducted in a hot environment by the present authors (Kubota et al., 2014b), as shown in Figure 6b; the weight of T‐shirts and trousers begin to increase at *w_sw_ ≈* 0.55 and *w_sw_* = 0.7–0.8, respectively.

(b) Standing posture by Kuno (1934).

**FIGURE 7:**
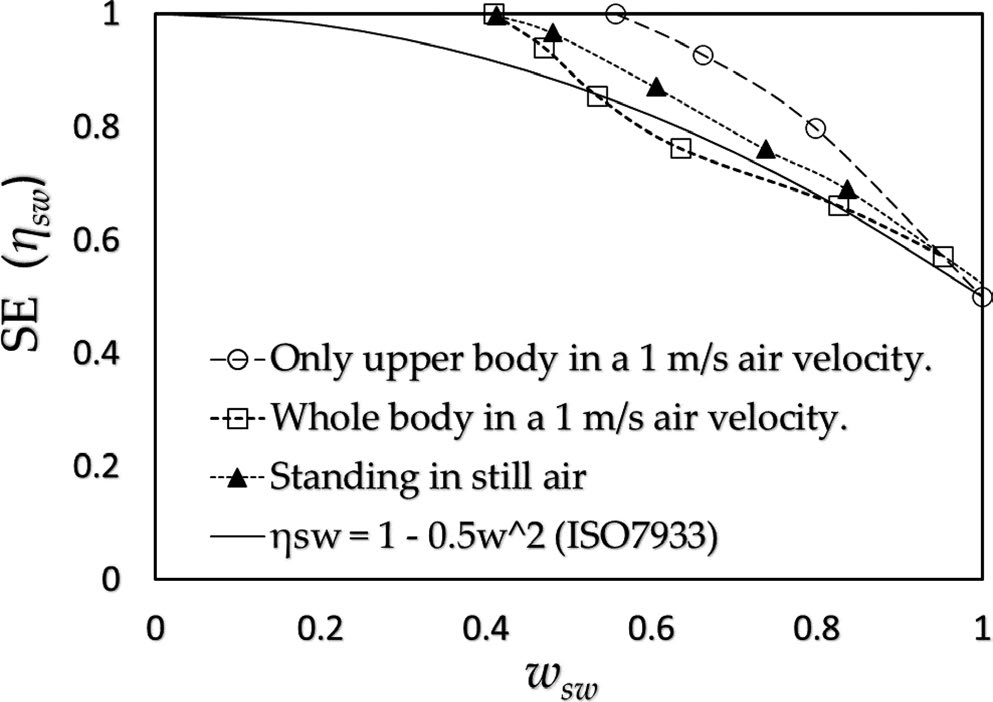
The SE for a subject in standing posture with the SR by Kuno (1934); the CE by Oliveira et al. for standing in still air (filled triangle); whole body in a 1 m/s air velocity (□); exposed only upper half body in 1 m/s (○).corresponds to the trunk area, and *w_dr_* ≈ 0.5 corresponds to the trunk, head, and thighs/pelvis regions (Kubota et al., 2014b)

The values of SE for a subject standing in still air was calculated for the values of SR by Kuno with the values of CE for a standing mannequin by Oliveira et al. The results of SE are shown in Figure 7, where the SEs are shown for a subject standing exposing a wind speed of 1 m/s to the whole or only the upper half body by which the distributions of CE were changed from that in still air.

(c) Stepping on and off a stool by Weiner (1945).

**FIGURE 8:**
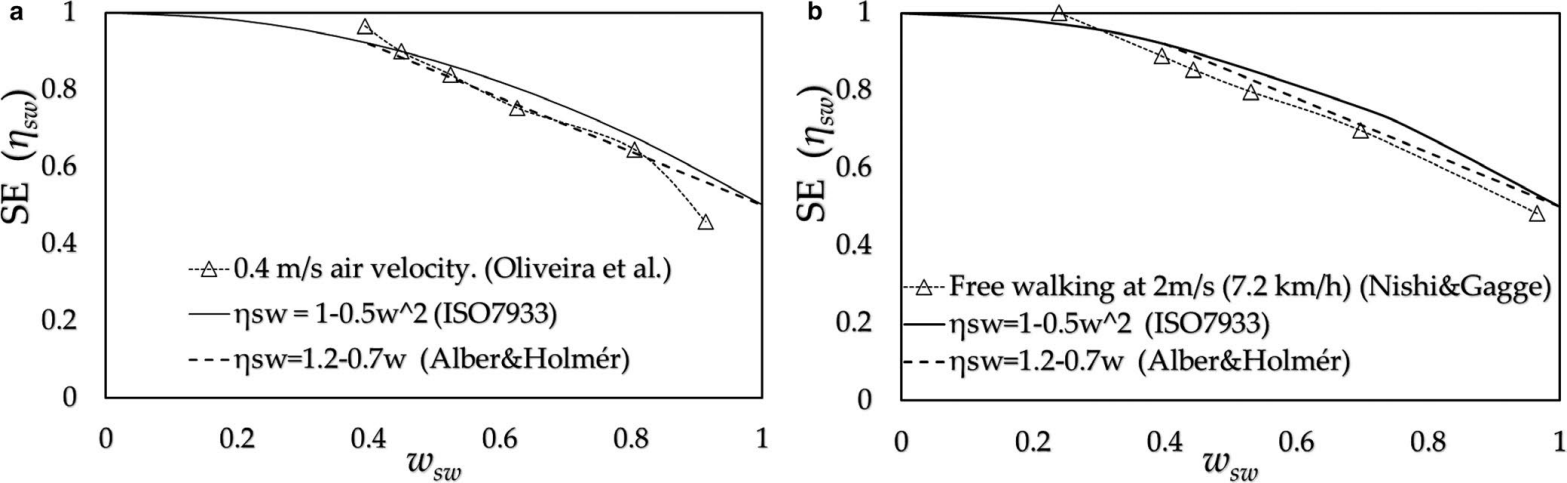
The results of SE for (a) subjects stepping on and off a stool given by Weiner (1945) combined with the CE for the posture of standing still in 0.4 m/s estimated from data given by Oliveira et al. (2014) (△); (b) for treadmill exercise of 55%VO_2max_ studied by Smith & Havenith (2011) combined with the CE for free walking at 2 m/s (7.2 km/h) by Nishi & Gagge (1970) (△)

Weiner conducted an experiment where subjects stepped on and off a stool; the SR results are shown in Figure 2 (The highest SR region is the chest for Weiner's data and the back for Smith & Havenith's data; further study is required to determine why the highest region differs). Figure 8a shows the results of SE. Figure 4b illustrates an example of the surplus of the SR over the CE at *w_v_* =1.1. We adapted the CE for the standing still posture assuming an airflow of 0.4 m/s, which corresponds to roughly the average moving speed of the body in Weiner's experiment (Appendix C); the values of *E_max_*_,_ *_i_*/*E_max_* (= *h_c_*_,_ *_i_*/*h_c_*) were obtained from data collected by Oliveira et al. (2014).

(d) Running on a treadmill by Smith & Havenith (2011).

Figure 8b shows the SE results for the SR obtained by Smith & Havenith (Figure 2) for a treadmill exercise of 55%VO_2max_ under 2 m/s frontal air velocity. The CE was estimated from those for free walking of 2 m/s speed (7.2 km/h) by Nishi & Gagge (1970) (Appendix C).

### 3.3 Improvement of the SE by arranging the distribution of air velocities over the body surface

**FIGURE 9:**
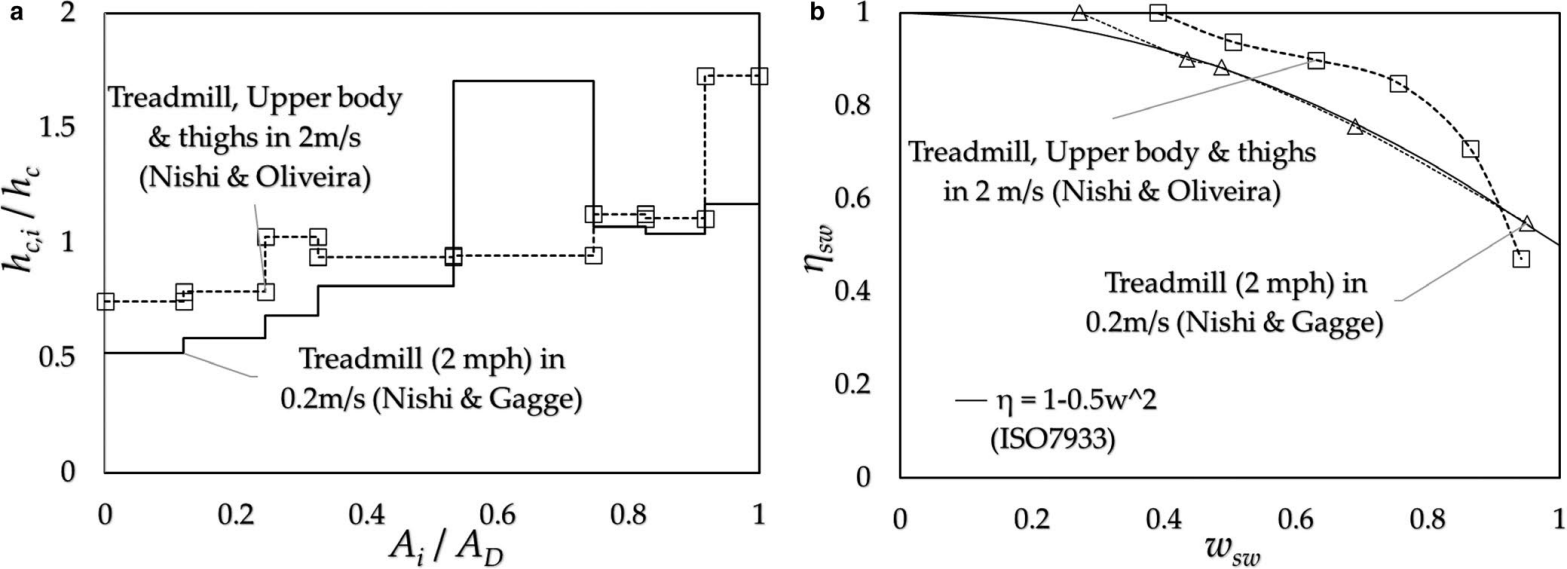
(a) Convective heat transfer coefficients.; treadmill exercise in a 2 m/s wind exposure of the upper body and thighs (*h_c_* =11.1) (□) and treadmill exercise at 2 mph in still air (*h_c_* =6.2) (Nishi & Gagge) (−). The regional unevenness of *h_c_*_,*i*_/*h_c_* (CE) in the case of upper body wind exposure becomes smaller than that for treadmill exercise in still air; (b) The predicted SE results for the SR estimated by Smith & Havenith combined with the CE; treadmill exercise with upper body wind exposure of trunk, head, arms and thighs (New‐SE) (□), treadmill exercise in still air (Nishi & Gagge) (△)

Based on the results described in Sections 3.1, 3.2, the SE value is determined by a combination of the regional SR and CE. Thus, making the air velocities higher at the lower CE areas (i.e., back and chest), can raise the relative values of CE to higher SR areas. Then, the SE values exceed those of wind with an even distribution of air velocities. We predict the SE, as an example, of subjects exercising on a treadmill at 2 mph simulating industrial/daily activities in an airflow of 2 m/s considering that their upper body above the knees is exposed to the wind; that is, uneven distribution of air velocities over the body surface. The values of CE (*h_ci_*/*h_c_*) for the upper body were estimated from data by Oliveira et al. (2014), whereas those for the lower legs were given by Nishi & Gagge (1970), as shown in Figure 9a. By exposing the upper body to the airflow, the values of SE shown in Figure 9b (hereafter, referred to as New‐SE) increased by approximately 13% compared with those without wind exposure.

### 3.4 Effect of the improvement of the SE on reducing the heat strain of a clothed subject

The MST was predicted as a typical heat strain indicator of a clothed subject by applying the New‐SE and the equation *η_sw_* = 1 – 0.5*wsw^2^* used in ISO7933 (hereafter, ISO‐SE). The effects of the New‐SE on alleviating the heat strain were assessed by comparing the MSTs predicted with New‐SE to those predicted with ISO‐SE. For the prediction of the MSTs, a previously developed human model (Kubota et al., 2014b) was used and the ISO‐SE was adopted. During the assessment, the ISO‐SE was replaced by the New‐SE. In the model, the wetted clothing effects were considered by introducing the concept of virtual dripping sweat rate (VDSR).

In the model, the VDSR was defined as the dripping sweat rate computed by replacing CE (*E_max_*) for a nude subject with that for the subject wearing dry clothing in Eq. (2) and Eq. (4). The VDSR is assumed to be the source of sweat that wet the clothing, thereby enabling a quantitative estimation of the wet clothing mass balance. As described in Section 3.1, the SEs are expressed by Eq. (15), and the values of the parameters of *a_dr_* and *b_dr_* are listed in Table 1.

**TABLE 1:** Parameters of *a_dr_* and *b_dr_* in Eq. (15) for the ISO**‐**SE and New‐SE

*w_sw_*	ISO‐SE 0.4–0.75	ISO‐SE 0.75–0.85	NEW‐SE 0.4–0.74	NEW‐SE 0.74–0.9
*a_dr_*	0.59	0.29	0.78	0.31
*b_dr_*	0.15	0.45	0.08	0.48

The conditions for clothed subjects were as follows: clothing of 0.42 clo (T‐shirts and trousers), metabolic rate of 3 and 4 met (1 met = 58.14 W∙m^−2^), mean air velocity of 0.85 m/s (*h_c_* =11.1), and air temperature equal to the mean radiant temperature. The procedure was as follows: (1) the adopted New‐SE function was as shown in Figure 9b and that of ISO‐SE was 1 − 0.5*w_sw_* ^2^; (2) the target MST (*t_sk_*) was set to *t_sk_* = 35.8°C, which was adapted as a temporary critical value of the MST assuming the results of Ref (Robinson et al., 1945) that cause the increase in the MST above 35.8°C to parallel the increase in the core temperature; and (3) the water vapor pressure in air *p_a_* (kPa) for *t_sk_* = 35.8°C was predicted at a given air temperature *t_a_* [°C].

**FIGURE 10:**
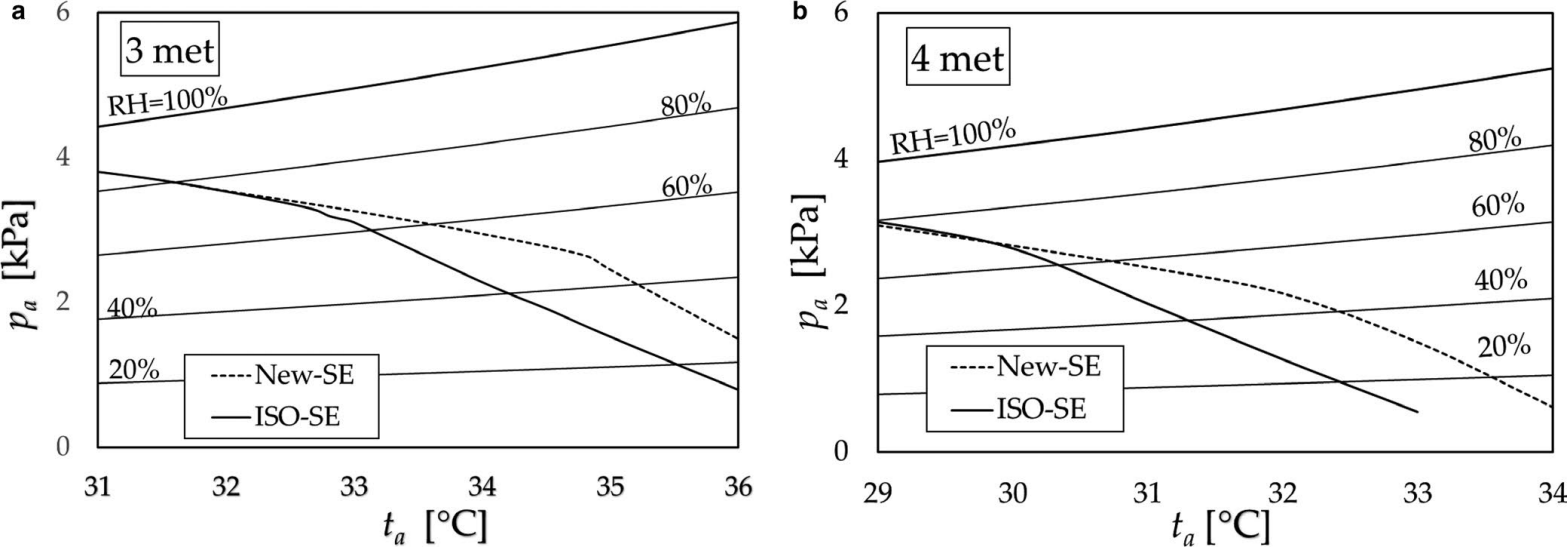
(a) Contours at *t_sk_* =35.8°C for a clothed subject at a 3‐met exercise predicted based on the New‐SE (dashed lines) and ISO‐SE (solid line); (b) Contours at *t_sk_* *=35*.8°C for a clothed subject at a 4‐met exercise predicted based on the New‐SE (dashed lines) and ISO‐SE (solid line)

The results are shown in Figure 10a,b as contour lines of *t_sk_* =35.8°C.

## 4 DISCUSSION

As shown in Figures 1 and 2, the SR at the trunk and head regions are higher than those at limbs; this implies that the physiological function for protecting the essential organs of the body from heat stress. In contrast, the CE has opposite character as shown in Figure 3, in which the lowest CE is found at the trunk (back or chest), and the highest CE is at the lower legs or hands, which will be determined by not the physiology but the physics (Kerslake, 1972) under the wind with air velocity *v*, forced convective heat transfer coefficient *h_c_* of the tube/globe of diameter *d* given roughly as *h_c_ ~* (*v*/*d*)^0.5^ (Nishi & Gagge, 1970); *d* of the trunk larger than those of limbs. In a still environment, the local natural convective heat transfer coefficient is inversely proportional to the height *x* from the floor, *h_c_ ~1*/*x^0^* ^.^ *^25^* (e.g., Incropera & DeWitt, 2002).

These characteristics on SR and CE provide the fundamental relationship between the SR and CE: the SRs at trunk/head are higher than those at limbs; however, the CEs at trunk/head are lower than those at limbs, which will result in the characteristics of the SE. Namely, this principle would roughly hold in daily life activities, such as standing, walking, running, because, in a wind or walking/running conditions, the air velocities against each region of the body are almost equal, and accordingly, the distribution of the CE (*h_c_*) are determined by the dimension (*d*) of each regions; however, when performing activities such as walking/running/cycling, the values of CE at lower limbs become higher due to their movements.

From the considerations above, the key point to improve the SE is to make the value of the CE at the trunk larger so that the largest surplus of the SR over the CE at the trunk becomes small.

In Section 3.1, an imaginary SE was analyzed for the two‐part‐pattern SR with uniform CE. Theoretically, the uniform CE (*h_c_*) could be attained by making the value of *v*/*d* constant for each region, for instance, when the air velocity *v_i_* at a body part *i* is controlled as *v_i_* = (*d_i_*/*d_min_*)∙*v_i_*_,_ *_min_* where *v_i_*_,_ *_min_* is the *v_i_* at the minimum *d* *(d_min_*): *d_min_* ≈ 0.1 m for lower legs/for‐arms, if fingers and toes are ignored (Nishi & Gagge, 1970). In practice, it would be difficult to distinguish *v_i_* at arms from that at the trunk because of their proximity to the trunk. If the distribution of CE is the same as that of SR, the SE becomes unity.

The theoretical result of Eq. (15) on the imaginary SE for the two‐part‐pattern was previously introduced by the present authors in Ref. (Kubota et al., 2014a) without the prior knowledge of *η_sw_*, which enabled the explicit derivation of the MST from the heat balance equation. The area of *a_HP_*/*A_D_* =0.31 for the cycle ergometer is nearly equal to the area of the “trunk and head.” Eq. (15) fits well the results obtained with the subject in the prone posture, which indicates that the subjects are likely to have a two‐part‐pattern SR and uniform CE distribution; the natural convective heat transfer coefficients at each region of the body in the prone position are close to each other, that is, small differences occur between vertical sizes of each region. The ratios *a_HP_*/*A_D_* (= *b_dr_*) and *S_LP_*/*S_w_* have values of 0.48 and 0.69, respectively, for the prone posture, which is larger than those for the cycle ergometer (0.31 and 0.54, respectively). This suggests that subjects in the prone posture sweat more uniformly than those exercising.

The difference in the SR distribution seems to be attributed to the postural influences on sweating related to muscle work; during exercise, such as on a cycle ergometer, the active thighs and other leg‐muscles need more blood flow, possibly because of the decrease in skin blood flow, which contributes to sweating (Wilmore et al., 2008). This causes a decrease in sweating on thighs and legs, resulting in a decrease in *S_LP_*/*S_w_*.

In Section 3.2, we calculated the SE based on SR and CE data at eight regions. At first, the results of a subject exercising on a cycle ergometer were compared and found to be considered close with the experimental results given by the same exercise by Alber‐Wallerström & Holmér (1985), and the equation of ISO‐SE as shown in Figure 6. This confirms the validity of the present method for predicting the SE, despite applying SR with an assumption which is similar to that for a subject running on a treadmill. It would be acceptable considering the similarity in the muscle work between the cycle ergometer and running on the treadmill, which burdens the lower limbs.

**FIGURE 11:**
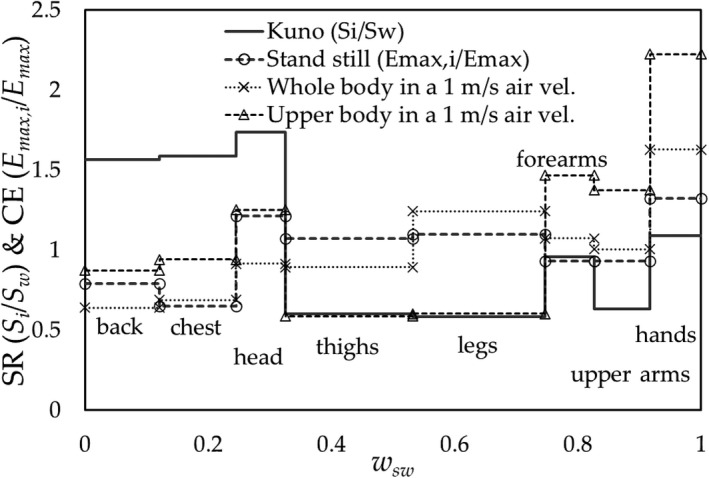
Distributions of the CE against the SR by Kuno for standing posture (−); CE in a still air (○); CE in a 1 m/s air velocity for whole body (×); and CE in a 1 m/s air velocity for upper half body (△)

Since the SE is determined by the relation between SR and CE, we can conclude at this stage that the equation of ISO‐SE, as an approximate equation of Alber & Holmér's experimental results, is only applicable for the specific combination of the distributions of SR and CE for a subject exercising on a cycle ergometer. However, as shown in Figure 7 (standing posture), Figure 8a (stepping on/off a stool), and Figure 8b (running on a treadmill), the results of SE are found to fall close to the lines given by Alber & Holmér and that of ISO‐SE. These results support the principle mentioned above: the relationship that exists between distributions of the SR (physiology) and CE (physics) would roughly hold in daily activities; further, as shown in standing posture in Figure 7, the SE in a still air (a) is slightly larger than that of ISO‐SE, and on the other side, that for the whole body in a 1 m/s air velocity (b) is slightly smaller than that of the case (a). These slight differences are connected to the amount of the surplus of the SR over the CE at the trunk, which roughly relates to the values of *CE_trunk_*/*CE_legs_*: (a) *CE_trunk_*/*CE_legs_* ≈ 0.74 (≈ (*x_legs_*/*x_trunk_*)^0.25^ by natural convection) which is larger than that of the case (b) *CE_trunk_*/*CE_legs_* ≈ 0.56 (≈ (*d_legs_*/*d_trunk_*)^0.5^ by forced convection) as shown in Figure 11. In contrast to the cases of (a) and (b), when the upper half body is in a 1 m/s air velocity (c), its value becomes *CE_trunk_*/*CE_legs_* ≈ 1.5 and the SE is considerably larger than that of ISO‐SE where the ISO‐SE cannot be applied.

In Section 3.3, aiming to reduce the heat‐associated strain, the improvement in SE was achieved by applying higher air velocity against the trunk/head accompanied by the arms. Empirically, in a hot environment, we applied an airflow against the trunk and head area. However, to create a strategy against the heat‐associated risks, we need to predict the physiological states of the subject in heat environments rationally for the evaluation of the heat strain, which requires identifying the function of the SE. For a higher air velocity over the trunk area, the SE values deviated from those given by the equation of ISO‐SE as this equation does not apply to every condition.

In Section 3.4, we examined the effect of the improvement of the SE to alleviate the heat strain of clothed subjects in hot environments by predicting the MST as a heat strain indicator using the previously developed human model in Ref.2. As shown in Figure 10a,b, for a given *p_a_* value, *t_a_* New‐SE contours (dashed lines) are higher than those of ISO‐SE (solid lines) at 3 or 4 met. For example, at *p_a_* *=* 2 kPa, *t_a_* computed based on the New‐SE at 4 met is approximately 32.2 °C, whereas that of ISO‐SE is approximately 31 °C, that is, the effect of the improvement of the SE to alleviate the heat strain of a clothed subject was identified quantitatively in terms of the MST prediction. Thus, we can conclude that the improved SE reduced the heat strain.

While some errors might be involved in the results described above due to the limitations arising out of the assumptions applied on the distributions of the SR and CE, and caused by the number of the regions of the body section and the characters of the subjects participating in experiments (only male but different states of physical fitness or acclimation in heat) in the data applied, the present authors believe that the essence of the conclusions described above remains the same.

## 5 CONCLUSIONS

The main conclusions are summarized as.

1. the SE is improved by applying airflow of higher air velocity to the trunk area of the body,
2. the equation *η_sw_* = 1 – 0.5*wsw^2^* in ISO7933 (ISO‐SE) does not apply to every condition, and
3. the SE improvement alleviates the heat strain.

To elucidate the SE via experiments requires time‐consuming work. We theoretically analyzed the SE of a nude subject by identifying the surplus of the regional SR over the regional CE by deploying data from the literature, and we improved the SE applied for the prediction of MST for the heat strain alleviation of clothed subjects. The main conclusions are as follows:

1. The fundamental characteristics on distributions of SR and CE was discussed: the SRs at trunk/head are higher than those at limbs due mainly to physiological reason; but the CEs are opposite due to physical reason, the CEs at trunk/head are lower than those at limbs, and this relationship could have a potential influence on the values of the SE for the daily activities.
2. The SE was calculated for an imaginary two‐part‐pattern SR (trunk/head vs. limbs) combined with the uniformly distributed CE, and an equation on the SE was derived as a function to be inversely proportional to the skin wetness, which was the same equation applied for the MST prediction.
3. The SE was calculated based on SR and CE data at eight regions for a subject participating in four activities: (a) cycle ergometer, (b) standing still, (c) stepping on and off a stool, (d) running on a treadmill. The results of SE for these activities were found to be close to those given by ISO‐SE, and this was attributed to the relationship underlining SR and CE as described in (1).
4. Aiming to improve the SE, the trunk and head regions were exposed to the higher air velocity, that is, an upper body exposure to the wind of 2 m/s. The acquired SE results became higher than those of ISO‐SE, suggesting that the equation of ISO‐SE does not apply to every condition.
5. Then, the effect of the improved SE was examined by predicting the MST of a clothed subject, and it was found that the SE improvement alleviates the heat strain.

The authors believe that the present models could contribute to the evaluation and alleviation of heat‐associated risks from an ever‐greater threat under the global warming trend. The accurate evaluation of the SE can be a cornerstone for the prediction of the heat strain of a human subject, which can lead to a new heat‐strain index expected to replace it with the empirical WBGT. Besides, in recent years, an increasing number of workers have been wearing jackets with fans to reduce the heat strain in a hot environment, where the trunk areas of the body are exposed to an airflow of higher air velocity (Kuwabara et al., 2020); the new approach of this paper could open the way to identify the characteristics of this garments rationally and to improve them with the predicted heat strain of MST. Further studies on the SR and CE for other conditions are desired for the identification of the SE.

## CONFLICTS OF INTEREST

The authors declare no conflicts of interest.

## APPENDIX A

The following data were used in this paper. SR data were deployed for four male subjects in the standing posture by Kuno (1934), three acclimated male subjects stepping on and off a stool of 1 ft at 12 or 24 steps/min by Weiner (1945), and nine male athletes running on a treadmill at two exercise intensities, 55% VO_2max_, by Smith & Havenith (2011). The number of measuring points at the skin surface was 20 (Kuno), 30 (Weiner), and 42 (Smith & Havenith). The CE data were deployed for a human subject exercising on a treadmill at 2 mph and cycle ergometer at 60 rpm and free walking at 2 m/s by Nishi & Gagge, a mannequin model at standing and walking at 45 steps/min in still air or some other air velocities by Oliveira et al.

## APPENDIX B

The relationship between the values of *E_max_*_,_ *_i_* and *h_c_*_,_ *_i_* are given by

(A1) $$E_{max,i}=LR\cdot h_{c,i}\cdot \left(p_{sk,i}‐p_{a}\right),$$

(A2) $$E_{\max}=LR\cdot h_{c}\cdot \left(p_{\mathrm{sk}}‐p_{a}\right),$$

where *E_max_*_,_ *_I_* and *E_max_* are the regional and mean CE at the wet skin surface in W∙m^−2^, respectively; *LR* denotes the Lewis number (=16.5) in K/kPa; *h_c_*_,_ *_i_* denotes the regional convective heat transfer coefficient in W∙m^−2^∙kPa^−1^; *p_sk_*_,_ *_i_* denotes the saturated water vapor pressure in air at the regional skin temperature *t_sk_*_,_ *_i_*; *p_sk_* represents the saturated water vapor pressure in air at the *t_sk_* (MST); and *p_a_* denotes the water vapor pressure in ambient air in kPa.

From the above equations, the following relation of CE is obtained assuming *p_sk_*_,_ *_i_*≒*p_sk_*.

(A3) $$\frac{E_{max,i}}{E_{\max}}=\frac{h_{c,i}}{h_{c}}.$$

## APPENDIX C

In Weiner's experiments, the subjects performed the work of stepping on and off a stool of 1 ft at 12 or 24 steps/min. We assumed that the moving speeds of the legs and trunk in Weiner's motion are nearly equal, and the relative air‐to‐body velocity (*v_w_* in m/s) was estimated by adding the average speed (*v_b_*) of the body movement to that of the environment (*v_e_*), which equaled to 0.2 m/s as

*v_b_* = 1 ft at 12 or 24 steps/min =0.30 m × 2 on/off / sin60° ×12 or 24 / 60 s^−1^ = 0.14 or 0.28 m∙s^−1^.

*v_w_* = *v_b_* + *v_e_* =0.14 or 0.28 + 0.2 = 0.34 or 0.48 ≈ 0.40 m/s.

For Smith & Havenith's experiments involving running on a treadmill in a 2 m/s frontal air velocity, we applied the CE values for a subject in the free walking at 2 m/s by Nishi and Gagge. The walking speed of 2 m/s corresponds to the frontal air velocity of 2 m/s. The existing differences between movements of running and walking such as up and down movement and the limb swing were ignored.
